# Performance of Maraging Steel Sleeves Produced by SLM with Subsequent Age Hardening

**DOI:** 10.3390/ma13153408

**Published:** 2020-08-02

**Authors:** Piotr Tyczyński, Zbigniew Siemiątkowski, Piotr Bąk, Krzysztof Warzocha, Mirosław Rucki, Tadeusz Szumiata

**Affiliations:** 1 Mapal Narzędzia Precyzyjne Sp. z o.o., ul. Partyzancka 11, 61-495 Poznan, Poland; Piotr.Tyczynski@mapal.com; 2 Faculty of Mechanical Engineering, Kazimierz Pulaski University of Technology and Humanities in Radom, Krasickiego Str. 54, 26-600 Radom, Poland; z.siemiatkowski@uthrad.pl (Z.S.); m.rucki@uthrad.pl (M.R.); 3 Yasa Motors Poland Sp. z o.o. S.K.A., ul. Wojska Polskiego 20, 39-300 Mielec, Poland; piotr.bak@yasa-motors.com (P.B.); krzysztof.warzocha@yasa-motors.com (K.W.)

**Keywords:** additive manufacturing, pro-environmental technologies, selective laser melting, porosity, fatigue, hyperfine magnetic field

## Abstract

In the paper, the researches on sleeves made out of maraging steel 1.2709 using selective laser melting (SLM) technology are presented. This additive technology is recognized as favorable for the environment, due to 100% use of material and durability of manufactured details. The fabricated sleeves underwent subsequent tests, in particular, microhardness, porosity and homogeneity of the material was examined before and after heat treatment and salt bath nitrocarburizing process. Two kinds of fatigue tests were performed. The first consisted of the typical sinusoidal alternating load, the other was the high pressure pulse load test close to the real work conditions. It is of high importance that the fatigue strength of the tested sleeves is considerably higher than that of the similarly produced details shaped as a standard samples for tensile stress. The Mössbauer spectrometry analysis of hyperfine magnetic field distributions proved that SLM did not change considerably the martensite structure at atomic level.

## 1. Introduction

The manufacturing costs of a given part increase along with the amount of material that needs to be cut until the finished product is complete. Additive manufacturing (AM) technology allows for a total reduction of material waste and does not require prepping semi-finished products, which usually generates additional costs. Manufacturing of net-shape functional components [1] or thin-walled components for aerospace industry [2], are good examples for benefits resulting from AM applications.

The main materials available for this process are: aluminum AlSi10Mg, cobalt chrome alloy Co28Cr6Mo, nickel alloy In718, maraging steel 1.2709, 316L stainless steel, 15-5PH stainless steel, titanium alloy Ti6Al4V, commercially pure titanium TiCP. The powders for further selective laser melting (SLM) process are produced using a gas atomization process and at the moment are predominantly generated as a by-product of other powder generation processes. As a result, powders are currently very expensive compared to other powders (namely aluminum powder used in the spray paint industry). A recent review of process parameters for SLM of Ti-6Al-4V was published by Shipley et al. [3], while Doubenskaia et al. [4] applied an integral analysis and presented results of a study on SLM process of intermetallic TiAl powder. Xia et al. [5] reported the results on researches on porosity evolution during SLM process of Inconel 718 alloy. Other team proposed X-ray CT method for discontinuity and porosity detection in the parts produced with SLM technology [6].

The present study is focused to analyze the maraging steel 1.2709 using SML technology that proved good results in tools fabrication [7]. The resulted material usually underwent further treatment in order to achieve desired shape or surface quality, as described by many researchers, e.g., [8] and [9]. Some scholars reported interesting results on general microstructure and mechanical properties of this material after SLM process and post heat treatment. It was reported that the quantity of austenite phase in maraging steel increased during aging treatment due to reversion of martensite to austenite [10]. Others examined its tensile, fracture, and fatigue strength and came to the conclusion that the overall mechanical performance, including the fatigue crack growth characteristics, of the SLM maraging steel after aging was similar to that of conventionally manufactured MS of the same grade [11]. It was found that the fatigue behavior of the SLM-processed austenitic steel AISI 316 L was strongly related to the building direction, leading to a reduction of fatigue life [12]. In case of bi-material specimens manufactured by SLM, the failure occurred in the material near the interface [13]. The influence of powder layer thickness on the quality of SLM printed parts was also studied [14]. It is expected that the fatigue behavior of the SLM-processed maraging steel will be dependent on defects, residual stresses, surface finish, geometry and size, layer orientation, and heat treatment, as it was demonstrated experimentally in case of Ti-6Al-4V alloy [15].

One of the most common processes applied to the maraging steel 1.2709 is the age hardening [16]. In the present study, we applied also a salt bath nitrocarburizing process afterwards, and performed analysis of structure and strength of the material. Apart from typical tensile tests two kinds of fatigue tests were made. The results are very important because in case of sleeves, one-sided tensile fatigue load is substantially different from the hoop stress in real work conditions, which was not addressed in previous publications.

## 2. Materials and Methods

The researches were aimed at a feasibility study on application of additive manufacturing, in particular SLM, to produce the sleeve type parts for hydraulic actuators, such as a fly-by-wire (FBW) actuator. The study involved tests on porosity, hardness, and fatigue strength of the final product material, as well as Mössbauer analysis of the components for rough powder sample and for the fabricated sleeve.

### 2.1. Characterization of the Examined Details

The test samples were made by AM technology (SLM), where metal powder plays a very important role. Quality of the applied metal powder has a major influence on mechanical properties of the produced detail, but it can also have impact on the build-to-build consistency, the reproducibility between AM machines, the production of defect-free components, the manufacturing defects on surfaces [17]. In the experiments, maraging steel (material 1.2709) powder produced by LPW Technology Ltd., Cheshire, UK, was applied.

**Figure 1:**
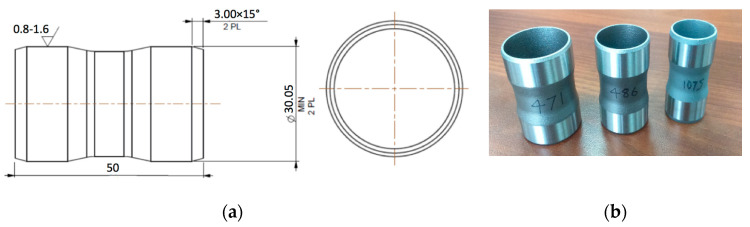
Details produced by AM technology for tests: (**a**) scheme; (**b**) photograph.

**Figure 2:**
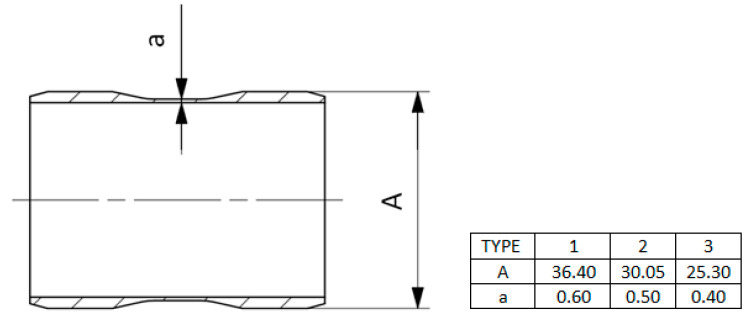
Dimensions of the three types of sleeves produced by AM technology for tests.

The scheme and general view of the tested sleeves are shown in Figure 1 and Figure 2 demonstrate the differences between the three types of the tested sleeves with wall thicknesses *a*_1_ = 0.6, *a*_2_ = 0.5 and *a*_3_ = 0.4 mm, respectively.

According to the producer’s specification, the chemical composition was as follows: Fe (balance), Ni (17–19 wt%), Co (8.5–9.5 wt%), Mo (4.5–5.2 wt%), Ti (0.6–0.8 wt%), Al (0.05–0.15 wt%), Cr and Cu (each ≤ 0.5 wt%), C (≤ 0.03 wt%), Mn and Si (each ≤ 0.1 wt%), P and S (each ≤ 0.01 wt%).

**Figure 3:**
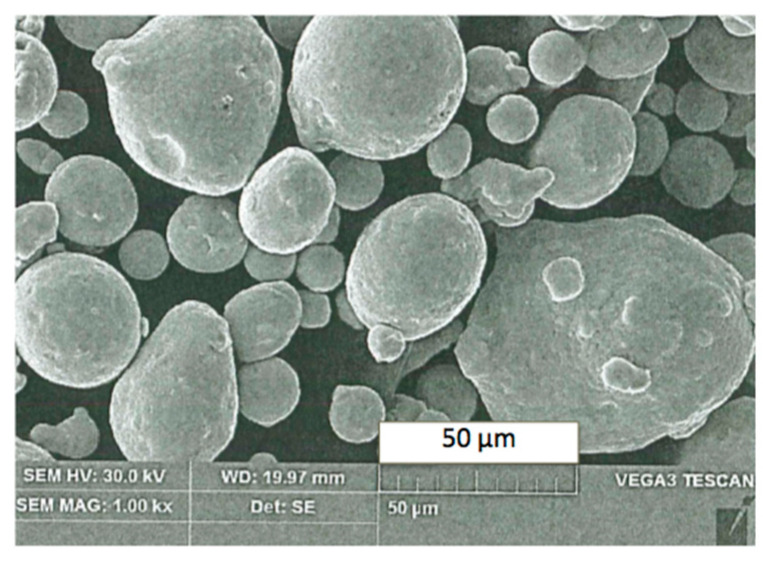
SEM image of the powdered steel 1.2709.

Density of bulk material 1.2709 is *ρ* ≈ 8.0–8.1 g/cm^3^. The initial material was powdered down to 43 μm particle diameter as a 90% prevailing fraction seen in scanning electron microscope (SEM) image in Figure 3.

Analyses were prepared and performed according to the standards EN ISO 945-1, ASMT A247, JIS G5502, KS D 4302, GB/T 9441. All the samples for further experimental researches were printed on a 3D printer ReaLizer SLM250, made by REALIZER, Borchen, Germany.

**Figure 4:**
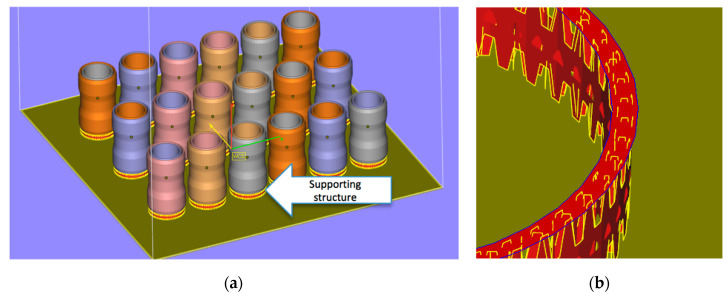
Arrangement of the samples in the 3D printer space: (**a**) overview; (**b**) example of supportive structure.

The process took place in protective gas atmosphere (argon), with maximum oxygen amount 0.3%. Base plate temperature was 80 °C. The samples were arranged on the plate as shown in Figure 4a, where supportive elements are shown in yellow red color and marked with arrow. These supporting structures shown in detail in Figure 4b allow for supporting steep overhangs and cantilevered sections of the model as it is built layer by layer. They are thin-walled, light and easy to remove structures, which help to avoid unnecessary material waste.

Among others, supportive elements are responsible for the proper heat removal. Thus, they have impact on the microstructure of the material.

### 2.2. Porosity and Microhardness

**Figure 5:**
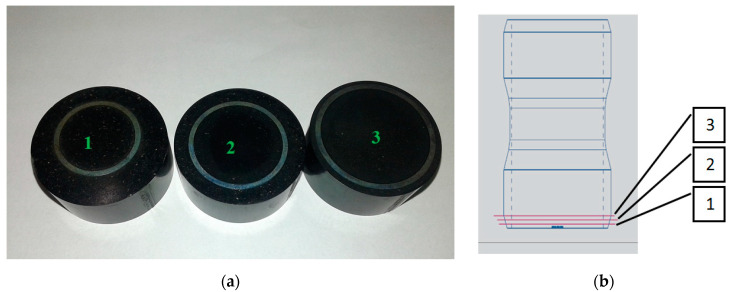
Metallographic test surfaces (**a**) and theirposition in the sleeve (**b**).

The research was aimed to analyze the porosity of material, so the percentage of pores in the volume of material produced by AM technology was measured. To achieve that, a series of samples underwent impregnation and polishing, as shown in the Figure 5.

Metallographic analysis was performed using the optical microscope Olympus BX51M (Olympus Corporation, Tokyo, Japan). The device allowed to analyze the images in white light, in reversed light, in polarized light, as well as in the interference contrast and in reflected light. The microscope was equipped with Koehler lighting system that ensures steady, stable and shadeless exposition of the sample surface, with smooth regulation in the entire range. Camera adapter enabled to make photos and acquisition, archiving and morphometric measurements. In order to measure percentage of the pores in the material structure, the dedicated software Olympus Stream Essentials was employed.

Hardness measurement of the 3D printed samples made of 1.2709 steel was performed using the device Qness Q250MS type (Qness Gmbh, Golling, Austria). It enabled to measure the Brinell hardness (DIN EN ISO 6506), Vickers hardness (DIN EN ISO 6506), Rockwell hardness (DIN EN ISO 6508) and Koop microhardness (DIN EN ISO 4545). The images of the test impressions are evaluated fully automatically using automatic brightness control and an autofocus camera to guarantee maximum process reliability. In the current research, the methods HV1, HV0.5 and HV0.1 were applied.

### 2.3. Fatigue Test Methodology

Apart from standard tensile testing procedure (PN-EN ISO 6892-1:2016-09, device Instron 3382), series of the experiments were performed in order to evaluate strength and fatigue resistance of materials. In the laboratory of Rzeszow University of Technology, Rzeszow, Poland, typical fatigue tests were performed. Sample made of the steel 1.2709 with AM technology had round intersections and dimensions in accordance with the standard ASTM E466-15. It was produced using SLM method with similar parameters as the examined sleeves, with layers added in the longitudinal direction. No surface machining was applied, only abrasive blasting was used to clean the surface before and after heat treatment. The exception was the surface of fixation to the device, which was machined but had no impact on the measurement results. The test was performed with the dedicated device INSTRON 8801 (Instron, Norwood, MA, USA) at room temperature.

The samples underwent the sinusoidal alternating load with frequency *f* = 3 Hz in conditions of one-sided tensile testing, where the tensions did not change their direction. Amplitude of the tensions is determined by the difference between their highest and lowest values, *σ_max_* and *σ_min_*, respectively:

(1) $$\sigma_{a}=\frac{\sigma_{max}-\sigma_{min}}{2}$$

Thus, the range of the tension’s variation was Δ*σ* = 2*σ_a_* = *σ_max_* − *σ_min_*. The cycle asymmetry factor was *R = σ_min_/σ_max_* = 0.05.

**Figure 6:**
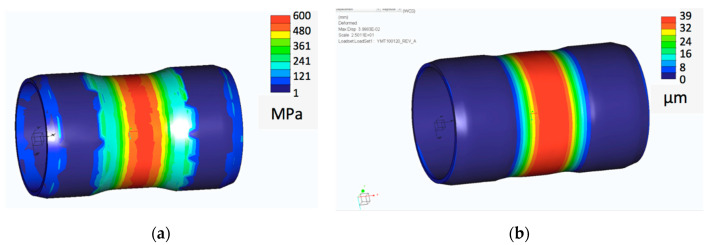
Examples of FEM simulation: (**a**) stress distribution; (**b**) deformations.

Since the layer-by-layer laser melting technology generates specific structure dependent on the shape and destination of the part, it reveals anisotropic fatigue performance dependent of the build direction [18]. Thus, another kind of fatigue resistance was related to the real work conditions of the sleeves produced with AM technology. Figure 6 presents the examples of hoop stress distribution and deformation in the tested details simulated by Finite Elements Method (FEM).

The hoop stress, also referred to as circumferential or tangential stress, is defined as follows [19]:

(2) $$\sigma_{h}=\frac{PD}{2a}$$

where *P* is design pressure, *D*—outside diameter, *a*—pipe wall thickness. In the experimental research, the samples were prepared with diameters *D*_1_ *=* 34.20, *D*_2_ = 28.00 and *D*_3_ = 22.88 mm, and respective wall thickness of *a*_1_ *=* 0.60, *a*_2_ = 0.50 and *a*_3_ = 0.40 mm.

**Figure 7:**
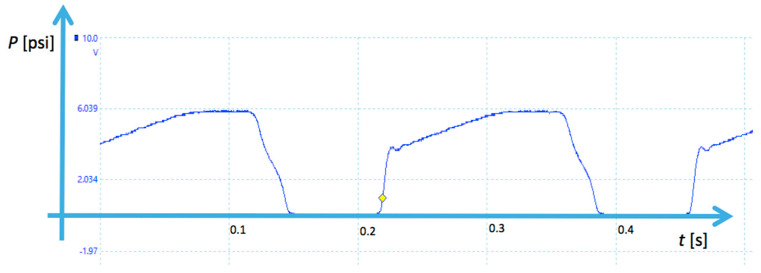
Example of pressure pulses registered by oscilloscope.

The experimental rig was built for the high pressure pulse load tests. The maximal pressure load obtainable in the test rig was 5000 psi (35 MPa), and the safety system stops the procedure when the sample is destroyed. The latter is indicated by the presence of oil outside the tested sleeve in the sink unit. During the work, the system generates pressure *P* pulses with period of ca. 0.2 s. The pressure transducer connected with oscilloscope enables to check the parameter of the pressure pulses. Example of the pressure indications registered by the oscilloscope is shown in Figure 7.

Working value of the pressure *P* = 35 MPa was reached by the regulator and set with accuracy of ±2%. The pressure should be checked and adjusted after the oil is heated. It was noted that the pressure dropped a little after some time, when the oil temperature became higher. Obtained pressure signal must be repeatable in its minimal and maximal values.

### 2.4. Mössbauer Spectrometer System, Fitting Methods and Samples Preparation

^57^Fe Mössbauer spectrometer for iron-containing phases investigations produced by Polon, Poland, and modernized at University of Technology and Humanities (UTH) in Radom, Poland, was arranged in vertical configuration. It was equipped with ^57^Co(Rh) γ-ray source and was operating in constant acceleration mode. Fitting of the Mössbauer spectra was performed by means of PolMoss v2.0 MulticoreTab software (by Tadeusz Szumiata, UTH, Radom, Poland) which is a package based on MS Excel and Solver optimizing module with gradient and genetic algorithms. This software was successfully utilized in the case both of the steel [20] and environmental samples [21]. Recently, it was updated for better performance of parallel calculations on multicore processors. PolMoss package provides a convolution of the Lorentzian baseline with Gaussian distributions of hyperfine parameters. This results in Voigt profile of fitted subspectra. The procedure of the convolution has been realized as numerical summation of finite number of Zeeman sextets scaled by discrete Gaussian distribution. Two samples for transmission Mössbauer measurements at room temperature were in the form of powders. The first one was just an initial powder, and the second one—shavings of sleeve filed with a ceramic file.

## 3. Results and Discussion

The obtained experimental results are presented and discussed in three groups: porosity and microhardness, fatigue strength and Mössbauer results, respectively.

### 3.1. Porosity and Microhardness

**Figure 8:**
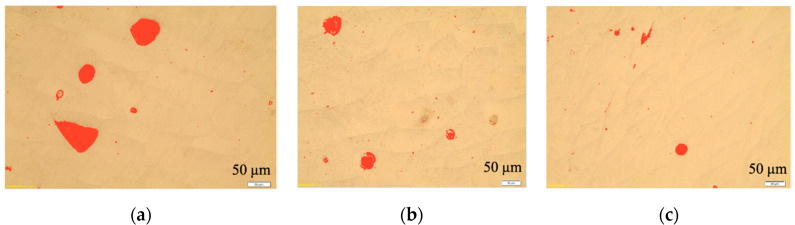
Analysis of the pores percentage in the respective intersections as explained in the Figure 5: (**a**) intersection 1; (**b**) intersection 2; (**c**) intersection 3.

**Figure 9:**
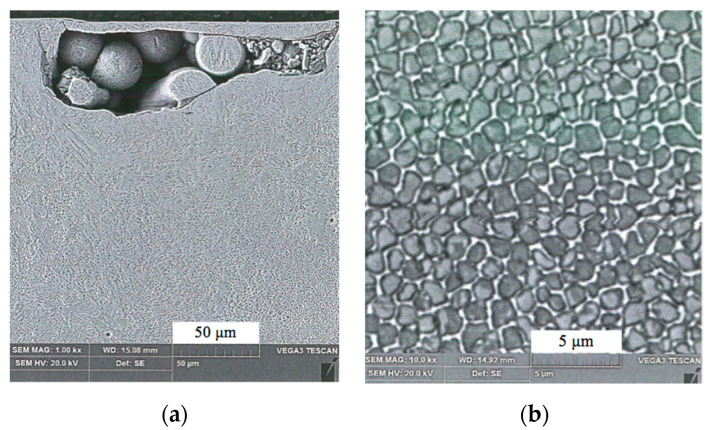
SEM images of microstructure: (**a**) example of the unmolten particles; (**b**) grained microstructure of the sample 3.

Figure 8 presents the example of the images obtained in the respective intersections 1, 2 and 3 explained in the Figure 5 above. The samples were etched with nital 1%, red stains represent pores. Analysis provided the information that the material in intersection 1 (Figure 9a) had 3.43% of the pores. This intersection corresponds with the layers adjacent to the supportive structure that were formed in the very beginning of the AM process. Subsequent analyzed intersections were formed later, as they are placed above the level 1. It is clearly seen in the Figure 9b,c, that number and dimensions of pores are smaller and smaller for the layers 2 and 3, which was confirmed by the measurement. The analysis revealed pores percentage of 1.24% and 0.56% for the intersections 2 and 3, respectively.

Moreover, in the material layers adjacent to the supportive structure that was to be removed, some unmolten powder particles were found. They were encapsulated together and surrounded with the material as shown in the photomicrograph in Figure 9a. On the other hand, the structure became stabilized above intersection 3, and revealed porosity close to 0.56%. Figure 9b presents an example of the microstructure in intersection 3 as it was explained in the Figure 5 above.

The microstructure seen in the Figure 9b exposes cellular structure with high uniformity of grains. It should be noted, however, that the bulk material grains do not reflect the size of “primary” powder particles. Martensite grains dimensions are between 0.5 and 1 μm, and they adhere closely to one another in the environment of retained austenite. At 2000× zoom, just few paths can be noted, where grains are not in close contact. Structural uniformity largely contributes to the material strength.

At the next stage of the research, the measurements of microhardness of core and surface layer were performed. In the experiments, three groups of samples were used. First group constituted the samples right after the 3D printing, with no additional operations that would improve uniformity of the structure. The second group consisted of the samples after dedicated heat treatment, specific for 1.2709 steel (so-called precipitation hardening or age hardening [22]). And to the third group belonged samples that after age hardening underwent also thermochemical modification by salt bath nitrocarburizing known as TENIFER process [23] that may have different impact on different materials [24]. The experiments were projected in the way that would enable to qualify the usefulness of abovementioned processes in further production of the specific details with AM technology considering their destinations, strength, fatigue resistance and other mechanical and physical properties. Especially in case of aerospace industry, importance of each part’s reliability is crucial.

In the experiments, precipitation hardening took place without any inertial gas shielding. The samples were heated in temperature 490 °C for 6 h, and then cooled down freely in normal ambient conditions. After that, samples from the third group were sent to the laboratory where complete salt bath nitrocarburizing process Quench–Polish–Quench (QPQ) was performed [25]. According to the procedure, the parts were first preheated to about 350 °C in air, and then put into so-called TF1 bath, consisting of alkali cyanate and alkali carbonate salt melt, at temperature 580 °C. Next, the samples were cooled down in a specially developed so-called AB1 bath at about 400 °C. Then, after being cooled to the room temperature, the samples were cleaned and polished, completing QP process (Quench–Polish), and underwent oxidative post treatment in the same salt melt and the same temperature finishing the entire QPQ process. The post-oxidation was aimed to restore some partial loose of the corrosion resistance after polishing.

**Figure 10:**
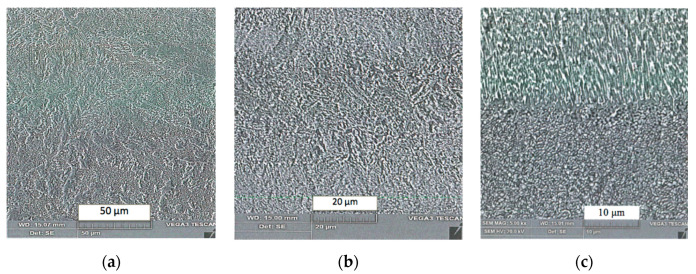
The SEM image of samples of different groups: (**a**) group1; (**b**) group 2; (**c**) group 3.

**Figure 11:**
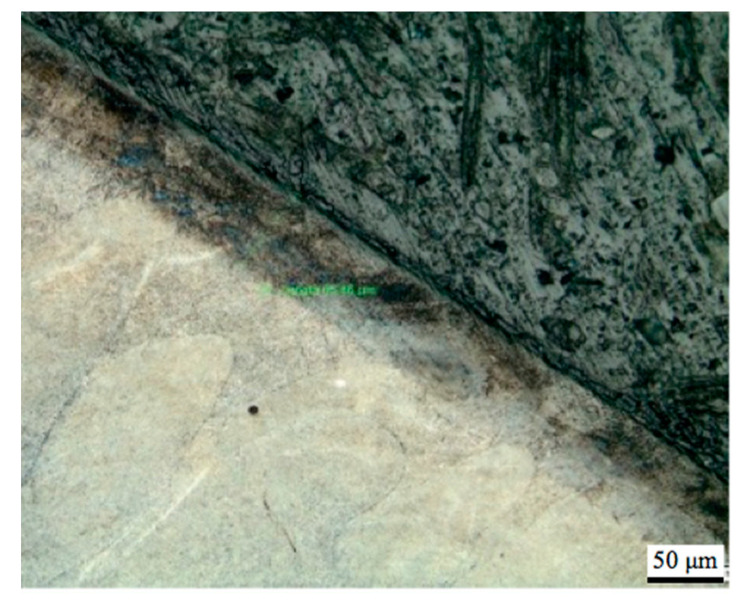
Photomicrograph of the sample of group 3.

Figure 10a–c presents examples of SEM image of the structures obtained in the sample that belonged to the respective groups 1, 2 and 3. In case of group 3, the surface layer modified by TENIFER process is clearly distinguishable in Figure 10c. In order to emphasize the modified layer, the samples were etched with nital, an example of the photomicrograph is shown in Figure 11.

The sample from the group 3 shown in SEM image in Figure 10c exposes grains prolonged in the direction perpendicular to the surface. No such effect can be seen both in the samples of group 1 (without heat treatment) and of group 2 (after age hardening but without TENIFER), shown in Figure 10a,b. Measurement performed with the photomicrograph provided information that the nitrogen diffusion layer was of depth ca. 68 μm. However, the actual nitrogen penetration depth and the respective nitriding hardness depth are pending to be considerably higher than the visible etchable dark area [25]. As it is demonstrated below, hardness measurement has confirmed substantial structural changes. The results are presented in the Table 1.

The analysis proved that the precipitation hardening process (group 2) and subsequent surface treatment (group 3) provide the improvement of hardness according to the available steel 1.2709 specification. However, it is important to make more detailed insight to the third group results, since it underwent two subsequent processes, heat treatment and ferritic nitrocarburizing.

**Figure 12:**
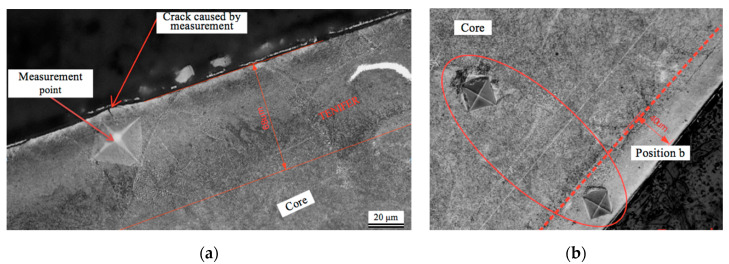
Photomicrograph of the microhardness measurement: (**a**) sample 1 with the measurement point *a*; (**b**) sample 2 with the measurement point *b*.

The nitrocarburised layer was subject of detailed measurement in order to determine its microhardness compared to the core material. The difficulties with microhardness distribution at the depth of the nitrocarburised layer rose because of its small thickness compared to the penetrator dents. For instance, when it was put too close to the edge of the sample, penetrator caused a crack seen in Figure 12a, which obviously affected the result of measurement. In case of sample 2, where visible nitrogen diffusion layer was just 40 μm wide, the penetrator dent covered ca. 75% of its width, as it is seen in Figure 12b. Table 2 presents the results of measurement.

As it is seen in the Table 2, hardness values HV0.5 for the TENIFER modified layer are 20–40% larger than the ones for the core material.

### 3.2. Fatigue Tests

First of all, it should be noted that the static tensile testing revealed very large range of variation between the samples in the elongation expressed by engineering strain ε. All the samples were produced with orthogonal scanning strategy, which reduced residual stress and porosity [26]. The hatch spacing was 0.050 mm and each subsequent layer was directed perpendicularly to the previous one. The laser power was 350 W and scanning speed was 1000 mm/s. After being printed, the samples for static tensile tests were heated in temperature 490 °C for 6 h, as it is recommended for maraging steels, to reach hardness 55 HRC which corresponds with ca. 595 HV.

In the Table 3, along with 0.2% offset yield strength R0.2 and maximum withstandable stress *R_m_*, there are presented the values of fracture A [%] are presented for different samples with respective “necking” or reduction in the diameter Z [%]. The fracture A varies between 1.2% and 6.9%, while diameter reduction Z between 5% and 21%. Maximum withstandable stress *R_m_* varies between 1287 and 1603 MPa, i.e., in range of 20% of its maximal value 1603 MPa.

The abovementioned variations can be attributed to the microstructure differences generated by the layer-by-layer melting technology. Despite any procedures aimed to make the microstructure uniform, it remained distinguishably layered. It might be expected that a degree of transverse strain anisotropy was likely to remain due to the fabrication history, which was reported for AM alloys [27]. Moreover, the presence of detrimental surface and subsurface defects could cause some scatter in the strength and fatigue test results [28]. Obtained 0.2% offset yield strength *R*_0.2_ and maximum withstandable stress *R_m_* of the AM samples of maraging steel may be considered repeatable in the satisfactory range.

**Figure 13:**
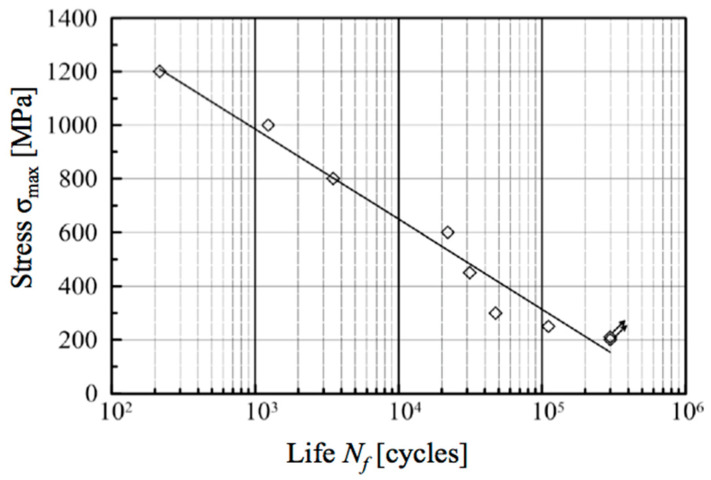
S-N logarithmic plot of one-sided tensile testing for fatigue strength of material 1.2709.

The results of the standard fatigue testing are shown graphically in Figure 13, where the logarithmic S-N plot is presented. The last two points with *σ_max_* = 210 and 200 MPa, and respective *σ_min_* = 10.5 and 10 MPa correspond with *N_f_* = 300,000 cycles, after which the sample did not break.

It should be noted that the maximal stress below 250 MPa did not cause destruction of the samples after 300,000 of cycles. Thus, it can be assumed that for the larger number of cycles than *N_g_* = 300,000, the fatigue strength of the steel 1.2709 in the one-sided tensile test conditions (*R* = 0.05) can be expected to reach 210–250 MPa.

These findings are important especially in comparison with the results obtained for the hoop stress measured in the sleeves. In that case, 300,000 cycles corresponded with stress *σ_max_* = 371 MPa (sample X0951). The stress values below 350 MPa were bearable even for million cycles without failure, and data from 6 samples confirmed it (each of two samples with wall thickness *a*_1_ *=* 0.6, two with *a*_2_ *=* 0.5 and two with *a*_3_ *=* 0.4 mm). The minimal stress bearable for the infinite number of cycles was calculated as *σ_min(inf)_*, and its value varied between 184 and 324 MPa. Thus, it can be noted that the fatigue strength of the sleeves made of the maraging steel 1.2709 using AM technology, is considerably higher than that of samples for tensile stress.

**Figure 14:**
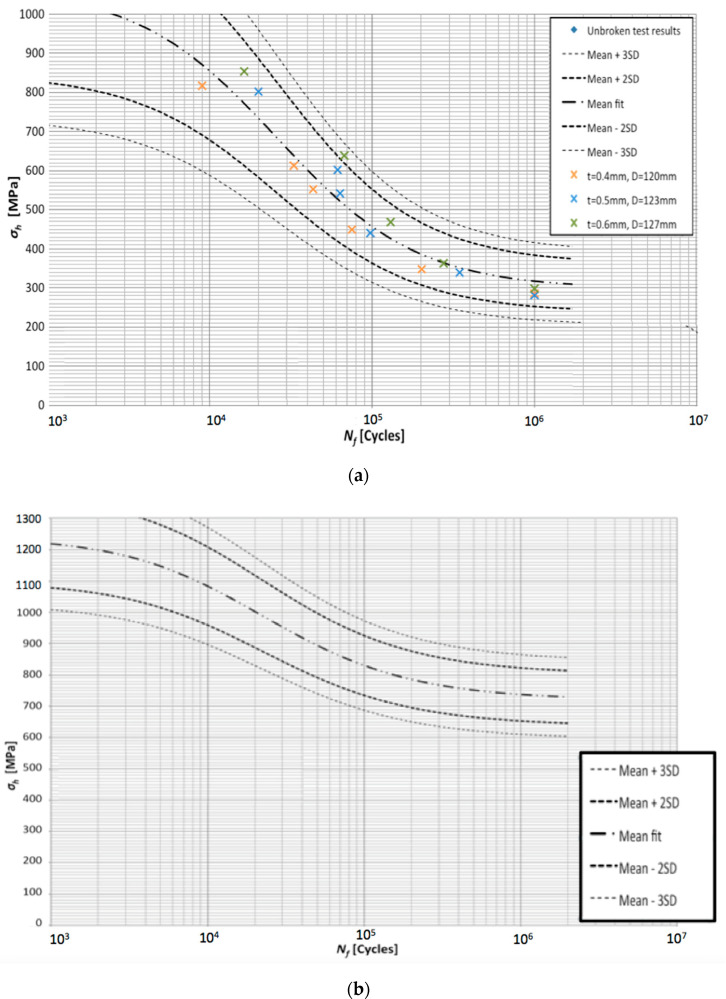
S-N logarithmic plot of hoop stress for fatigue strength of the tested sleeves, material 1.2709 with different porosity: (**a**) 0.4–0.6%; (**b**) 0.05–0.1%.

Figure 14 presents S-N graph with statistical analysis for the sleeves after heat treatment at 490 °C during 6 h. The porosity of the material was 0.4–0.6%. The double-dotted curve represents the mean fit curve, while the dashed curves correspond with the standard deviation ranges 2SD and 3SD.

As it can be expected, larger percentage of the pores in the material leads to its weakening, perhaps because of more easy crack propagation. Higher porosity (0.4–0.6%) provided hoop stress at average *σ_h_* = 930 MPa, 490 MPa, and 300 MPa after 10^4^, 10^5^, and 10^6^ cycles, respectively, while smaller porosity (0.05–0.1%) ensured respective values *σ_h_* = 1100 MPa, 830 MPa, and 730 MPa. In case of 10^6^ cycles, the hoop stress for smaller porosity is more than twice higher than in case of more porous material.

It is noteworthy that in comparison with tensile S-N fatigue plot, the hoop stress is not linear in logarithmic scale. Both higher fatigue strength and curvature may be attributed to the microstructure of the produced details. Since the material was added layer-by-layer, its strength in different directions can be expected to be different. The damage of the strained sample always takes place along the plane between the layers, which may explain smaller bearable stresses than in case of hoop stress directed perpendicularly to them. The detailed study of the microstructure impact is under way.

### 3.3. Mössbauer Spectrometry

**Figure 15:**
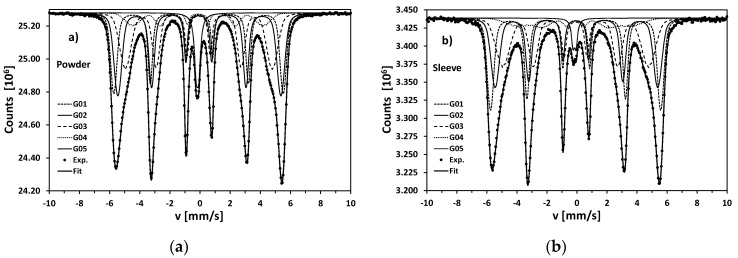
Room temperature transmission Mössbauer spectra for the initial powder and ready sleeve: (**a**) initial powder; (**b**) SLM fabricated sleeve. The components G01–G04 represent martensite while G05 does retained austenite.

Mössbauer spectrometry, as a very common technique in the case of various kinds of steels [20,29,30,31,32], has been utilized in order to determine iron-bearing phases. Transmission Mössbauer spectra (TMS) collected at room temperature are presented in Figure 15a,b for the initial powder and 3D printed sleeve, respectively. The spectra were fitted with a set of 5 Voigt-like sextets. G01-G04 components were utilized in order to describe the smeared sub spectrum very characteristic for martensite in maraging steel [29,30].

**Figure 16:**
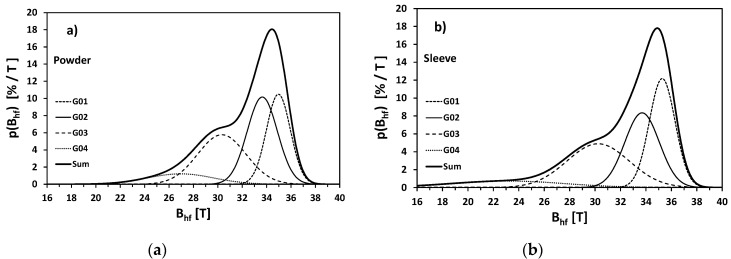
Distributions of hyperfine magnetic field corresponding to the martensite phase in the respective samples: (**a**) initial powder; (**b**) SLM fabricated sleeve.

According to the Mössbauer data for binary alloys [33] and considering the elemental content of the investigate steel, the subsequent components could be interpreted as different Fe sites (surroundings) in martensite structure (Table 4): with one Co or Ni atom in the first coordination zone (G01), only Fe atoms as the nearest neighbors (G02), one Mo or Ti atom in the first coordination zone (G03), several Mo or Ti atoms as nearest neighbors (G04). These components correspond to four Gaussian distributions of the magnetic hyperfine field *B*_hf_ reproducing quasi-continuous, resulting distribution for whole martensite phase (Figure 16).

Each component is characterized by mean value of hyperfine magnetic field distribution *B*_0_ (from the range 23–35 T) and the width of this distribution *DB*_hf_ (i.e., standard deviation). The values of these quantities are in general very similar for the rough powder and SLM 3D printed sleeve (Table 4). The most pronounced difference is visible only in the lower mean value of G04 component and in significantly greater width of the distribution. One can also see a reasonable agreement of in values of other hyperfine parameters i.e., isomer shift (δ, relatively to α-Fe calibration foil) and quadrupole splitting (Δ*EQ*) as well as in the contributions of the G01–G04 components. In general, both δ and Δ*EQ* are relatively low pointing to the structure of martensite not much deformed with respect to the body centered cubic lattice of α-Fe.

Metrologically, more precise comparison would be provided by the analysis of mean values, widths and asymmetries of the whole distributions of the hyperfine magnetic field *P*(*B*_hf_) corresponding to the martensite phase in rough powder and SLM 3D printed sleeve. The respective parameters are collected in Table 5.

The mean values <*B*_hf_> differs very slightly (32.41 T − 32.24 T = 0.17 T) i.e., about 0.5%. Such small difference is a strong proof that the local atomic ordering is very similar in both samples despite significant difference in morphology and metallographic microstructure. Nevertheless, Mössbauer technique was able to detect such subtle changes in structure, because <*B*_hf_> were determined with very good, spectroscopic precision 0.01–0.02 T, i.e., better than 0.03–0.06%. More pronounced difference exhibit the widths of hyperfine magnetic field distributions (defined as a square root of the variance—i.e., standard deviation). In the case of sleeve this width increased by more than 35%. This feature is easy visible directly in Figure 16 and points to the fact that though majority of SLM material reveals almost the same atomic ordering like in the initial powder, in some areas are the clusters of higher concentration of Mo, Ti, Al or Cu. Additional characteristics of the *P*(*B*_hf_) shape is Pearson-Fisher asymmetry coefficient:

(3) $${\tilde{\mu }}_{3}=\int P\left(B_{\mathrm{hf}}\right){\left[\frac{B_{\mathrm{hf}}-\langle B_{\mathrm{hf}}\rangle }{\sigma \left(B_{\mathrm{hf}}\right)}\right]}^{3}dB_{\mathrm{hf}}$$

which is one of the possible measures of skewness. For both samples it takes negative values, because *P*(*B*_hf_) distribution is widened towards left side. Nevertheless, the absolute value of ${\tilde{\mu }}_{3}$ is almost 70% higher for sleeve than for powder. It means that asymmetry coefficient of hyperfine magnetic field distribution is extremely sensitive parameter to subtle changes of atomic ordering in martensite.

The last component in the Mössbauer spectra (G05) is a doublet attributed to the retained austenite. Its content in the rough powder was about 5.6%, and in 3D printed sleeve—significantly less—c.a. 2.4%. It means, that SLM process facilitates austenite-martensite transformation. Retained austenite in sleeve is characterized by higher value of quadrupole splitting Δ*EQ*—possible because of stronger deformation of γ-Fe fcc crystalline structure after SLM. It worth underlining, that in the Mössbauer spectra corresponding to iron oxides were detected (sextets of hyperfine field close to 50 T)—nor in the initial powder nor in the sleeves. It is a good proof for the high quality of maraging steel powder and for the effectiveness of argon protective atmosphere during SLM process.

## 4. Conclusions

The researches were aimed to the strength analysis of the maraging steel details produced out of powder with SLM additive technology. Microsegregation of solute elements caused cellular structure of steel, i.e., martensite inside the cells and retained austenite in intercellular spaces. These features attributed to the strength of the 3D-printed sleeves.

Microstructure of the obtained details revealed differentiated porosity with higher pore percentage at the base, which became stabilized at some reasonably small height. In the material layers adjacent to the supportive structure, some unmolten grains of powder were found, encapsulated into large pores. These structural discontinuities at edges, however, did not affect the strength of the produced details or their fatigue resistance, because the main stress load occurs in the central area of the sleeve. It was confirmed, however, that the overall porosity of the material had direct impact on its strength. Other important finding was made using the analysis of hyperfine magnetic field distributions with transmission Mössbauer spectrometry. It revealed that the applied SLM technology does not change noticeably the martensite structure at atomic level. However, a more than two-fold decrease in the retained austenite content was observed. Moreover, no traces of iron oxides were detected, what is a proof of the efficiency of the argon protective atmosphere.

Some differences between obtained fatigue strength values may be explained considering the layer structure of the SLM-produced details, which require further detailed study. The bearable hoop stress is somewhat larger than the maximum withstandable stress *DB*_hf_, which also corresponds with real work conditions. Namely, the tested sleeves produced by AM technology are destined to undergo the pressure load, not strain. The difference takes place despite the uniform conditions of the SLM process and subsequent procedures aimed to unify the material structure.

The analysis proved that the precipitation hardening process increased microhardness of sleeves from ca. 350 HV up to ca. 400 HV, and subsequent surface treatment increased it further above 500 HV (hardness of modified layer was above 600 HV). The treatment had also some positive impact on the grain structure of the material increasing its strength.

The results proved that in case of sleeves, one-sided tensile load is substantially different from the one in real work conditions. The fatigue strength of the sleeves made out of the maraging steel 1.2709 using AM technology, is considerably higher than that of the sleeves shaped as a standard samples for tensile stress. It can be attributed to the directed material structure shaped during 3D scanning.

## Figures and Tables

**Table 1:** Microhardness measurement results.

No.	Group 1 (Without Heat Treatment) Measurement Method HV0.1	Group 2 (after Age Hardening but Without TENIFER) Measurement Method HV0.1	Group 3 (after Age Hardening and TENIFER) Measurement Method HV0.1 (0.025 mm from Surface)
1	344 HV	420 HV	560 HV
2	355 HV	397 HV	554 HV
3	331 HV	389 HV	508 HV
Standard deviation	9.809	13.140	23.230

**Table 2:** Microhardness measurement results in different points.

Sample No.	Thickness of Modified Layer [µm]	Hardness of Modified Layer in Its Middle [HV 0.5]	Hardness of Core (160 μm from Surface) [HV 0.5]
Sample 1 point *a*	68.08	603	475
Sample 2 point *b*	44.51	698	432
Sample 3 point *c*	65.86	659	478

**Table 3:** Results of static tensile testing of 1.2709 steel samples (Instron 3382).

Sample No.	*R*_0.2_ [MPa]	*R_m_* [MPa]	*A* [%]	*Z* [%]
1	1317	1432	2.9	18
2	1359	1450	5.1	21
3	1445	1530	4.4	21
4	1309	1397	1.2	5
5	1530	1603	2.8	13
6	1338	1417	6.8	10
7	1268	1336	2.8	5
8	1259	1347	6.0	6
9	1204	1287	4.8	8
10	1442	1514	6.9	13

**Table 4:** Contributions and hyperfine parameters of fitted Mössbauer spectra components for initial powder sample and the sleeve (the recognition of martensite-like and austenite-like components according to [29,30,31,32,33,34,35]).

Component	Phase	*P* [%]	δ [mm/s]	Δ*EQ* [mm/s]	*B*_0_ [T]	*DB*_hf_ [T]
Powder						
G01	martensite Fe-(Co,Ni) sites	25.8(3)	0.035(1)	−0.051(3)	34.97(2)	0.98(1)
G02	martensite Fe-Fe sites	32(1)	0.047(2)	0.038(4)	33.65(2)	1.24(1)
G03	martensite Fe-(Mo,Ti) sites	29(2)	0.022(1)	0.009(2)	30.33(1)	1.99(6)
G04	martensite Fe-n(Mo,Ti) sites	8.3(1)	−0.035(2)	0.011(8)	27.0(3)	2.7(1)
G05	retained austenite	5.6(1)	−0.059(1)	0.168(5)	-	-
Sleeve						
G01	martensite Fe-(Co,Ni) sites	30(3)	0.036(3)	−0.041(3)	35.28(7)	1.01(5)
G02	martensite Fe-Fe sites	28(3)	0.050(1)	0.043(0)	33.7(1)	1.36(8)
G03	martensite Fe-(Mo,Ti) sites	30.0(1)	0.017(2)	0.012(2)	30.23(1)	2.5(1)
G04	martensite Fe-n(Mo,Ti) sites	8.2(1)	−0.045(5)	−0.07(5)	23.11(8)	5(1)
G05	retained austenite	2.4(3)	−0.056(2)	0.21(3)	-	-

**Table 5:** Comparison of magnetic hyperfine field distribution *P*(*B*_hf_) parameters corresponding to martensite component (mean value, width—as square root of variance and Pearson–Fisher asymmetry coefficient).

Sample/*B*_hf_ Distribution	Mean <*B*_hf_> [T]	Width *σ*(*B*_hf_) [T]	Asymmetry ${\tilde{\mu }}_{3}$
Powder	32.41 ± 0.01	2.98 ± 0.03	−0.99 ± 0.04
Sleeve	32.24 ± 0.02	4.03 ± 0.18	−1.67 ± 0.25
